# Semen quality and reproductive hormone levels in men from Southern Spain

**DOI:** 10.1111/j.1365-2605.2010.01131.x

**Published:** 2012-02

**Authors:** M F Fernandez, I Duran, N Olea, C Avivar, M Vierula, J Toppari, N E Skakkebæk, N Jørgensen

**Affiliations:** * Laboratory of Medical Investigations, San Cecilio University Hospital, University of GranadaGranada, CIBER Epidemiología y Salud Pública (CIBERESP), Spain; † Integrated Area of Biotechnology, Hospital Poniente El EjidoAlmeria, Spain; ‡ Department of Physiology and Paediatrics, University of TurkuTurku, Finland; § University Department of Growth and ReproductionCopenhagen, Denmark

**Keywords:** normal men, reproductive hormones, semen quality, Spain, sperm concentration

## Abstract

In North European countries, a significant difference in semen quality among young men has been shown. Men from the western countries, Denmark, Germany and Norway, have lower semen quality than men from the eastern countries Finland, Estonia and Lithuania. Similarly, men in the western countries have a higher risk of testicular cancer. According to the testicular dysgenesis syndrome (TDS) concept that suggests a link between risk of impaired semen quality and increased risk of testicular cancer, Spanish men would be expected to have a semen quality at a normal level because of their very low testis cancer risk. We therefore investigated 273 men from the Almeria region in the Southern Spain to test this hypothesis. The men delivered semen samples, underwent physical examinations, had a blood sample drawn and provided information on lifestyle and reproductive health parameters. The investigations took place from November 2001 to December 2002. Adjusting for effects of confounders, the median sperm concentration and total sperm count were 62 (95% confidence interval 47–82) million/mL and 206 (153–278) million, respectively. The median numbers of motile and morphologically normal spermatozoa assessed according to strict criteria were 59% (57–62%) and 9.4% (8.6–10.0%), respectively. The median total testosterone and calculated free androgen index were 28 nm (26–30) and 95 (88–103), respectively. Assuming that the investigated men, to a large extent, are representative of the population of young men the Southern Spain, the results show that these have normal semen quality and reproductive hormone levels as expected in a population with a low incidence of testicular cancer.

## Introduction

The publication by Carlsen *et al.* (1992) that utilized historical data indicated an overall decrease in human semen quality worldwide. A re-analysis found this decrease to be more pronounced for Europeans than for Americans (Swan *et al.*, 2000). These publications also indicated differences in semen quality between men from different countries, which were corroborated by studies of fertile men (Auger *et al.*, 1997; Jørgensen *et al.*, 2001; Swan *et al.*, 2003). Prospectively designed, standardized cross-sectional studies from northern European countries have also confirmed these differences. Young men from the general populations in Denmark, Germany and Norway have been shown to have worst semen qualities, Swedish and Lithuanian men, intermediate, and Finish and Estonian men, best (Jørgensen *et al.*, 2002; Punab *et al.*, 2002; Richthoff *et al.*, 2002; Paasch *et al.*, 2008). Interestingly, similar geographical trends have been observed for testicular cancer with men from Denmark, Germany and Norway having the highest incidence rates (Huyghe *et al.*, 2007; Chia *et al.*, 2010). It has been suggested that an impaired development of foetal testes might lead to increased risks of cryptorchidism, hypospadias, decreased spermatogenesis or testis cancer (Jacobsen *et al.*, 2000; Skakkebæk *et al.*, 2001). The concept links the pathogenesis of the four disorders together, but does not imply that all affected men develop all four symptoms. The least affected may merely have a slightly reduced spermatogenic capacity, and only the most severely affected may present all symptoms (Jørgensen *et al.*, 2010).

Registry data have shown Spanish men to have lower testicular cancer incidence rates than men from most other European countries, although increasing rates have been noted (Bray *et al.*, 2006; Llanes González *et al.*, 2008). Thus, Spanish men would be expected to have a semen quality at a normal level. We therefore undertook a study of semen quality of normal young men from the Almeria province in the Southern Spain to test this hypothesis.

## Materials and methods

The investigation procedures described below were the same as those of the previously published European studies (Jørgensen *et al.*, 2002; Punab *et al.*, 2002; Paasch *et al.*, 2008) except for the invitation procedure. All information and investigations were undertaken at Poniente Hospital in Almeria except for the initial invitation to the study. The study was approved by the Poniente Hospital Human Subjects Ethical Committee.

### Invitation procedure

We informed about the study via television, radio and newspaper, and aimed at undergraduate populations in the study area, and we informed directly about the study at the University of Almeria ‘Open Days’ at the beginning of the academic course.

### Study subjects

Three hundred and fifty men responded to the information and were found eligible. These men were invited for further oral and detailed written information at the study centre. Following that 280 volunteered, including delivery of one semen sample. Among the 280 men, seven failed to deliver a semen sample, and the final study population comprised 273 men.

The inclusion criteria were that the mothers of the men were born in Spain, the men were born in the Almeria province (Andalucia region, Southern Spain) and living there at time of participation in the study. Furthermore, the men should be without direct knowledge of their testicular function. Thus, previous conception, previously or currently deliberately trying to conceive, or previously had semen analysed were criteria for exclusion.

At the information at the study centre, the men were given a questionnaire to fill in, instructed to preferably abstain from ejaculation for at least 48 h prior to producing the semen sample for the study, and an appointment for the investigations approximately 1 week later was made. Failure to comply with the request for ejaculation abstinence period was not a reason for exclusion. On the day of attendance, the men returned the completed questionnaire, underwent a physical examination, provided a semen sample and had a blood sample drawn. The investigations took place from November 2001 to December 2002. Participants received 20 Euros for their contribution.

### Questionnaire

Prior to the study, the questionnaire, originally in English, used in Denmark, Norway, Finland and Estonia (Jørgensen *et al.*, 2002), was translated into Spanish by professional translators. A back-translation to English was performed to check for discrepancies and for correction. The questionnaire included information on age and previous or current diseases, including any known history of fertility. To ensure quality of the information regarding previous conditions, the men were asked to complete the questionnaire – if possible – in collaboration with their parents.

### Physical examination

The participants had their testes size measured by use of a Prader orchidometer, presence of varicocele or other scrotal abnormalities and the Tanner stage of pubic hair evaluated. Body weight and height were measured, and body mass index (BMI) was calculated as weight in kilograms divided by squared height in metres. One physician did all the physical examinations.

### Semen samples

The participants were instructed to collect the semen samples by masturbation and ejaculation into a wide-mouthed plastic container. Majority of participants produced the sample at the study centre (80%). The remaining collected the sample at home and brought it to the laboratory within 1 h after ejaculation, protecting the sample from extreme temperatures. The actual abstinence period was assessed as the time between current and previous ejaculation as reported by the individual participant.

Semen volume was estimated by weighing the collection container and subtracting the predetermined weight of the empty container, assuming that 1 mL = 1 g. For sperm motility assessment, 10 μL of well-mixed semen was placed on a clean glass slide that had been kept at 37 °C and covered with a 22 × 22 mm coverslip. The preparation was placed on the heating stage of a microscope at 37 °C and immediately examined at ×400 magnification. The spermatozoa were classified as either motile or immotile (WHO, 1999) to report the percentage of motile spermatozoa. For the assessment of the sperm concentration, the samples were diluted in a solution of 0.6 m NaHCO_3_ and 0.4% (v/v) formaldehyde in distilled water. The sperm concentration was subsequently assessed using an improved Neubauer haemocytometer. Only spermatozoa with tails were counted. Smears were prepared for morphological evaluation, Papanicoulaou stained and finally assessed according to strict criteria.

One person did all the semen analyses in Spain, except morphology assessment for which the smears were sent to the andrology laboratory in Turku, Finland, for evaluation according to strict criteria (Menkveld *et al.*, 1990). However, some of the morphology slides were also assessed in Almeria according to the same criteria.

### External quality control of assessment of sperm concentration

A quality control programme to monitor inter-laboratory variation in sperm concentration assessment was coordinated by the University Department of Growth and Reproduction at the Rigshospitalet in Copenhagen (Denmark). Blinded undiluted fresh semen samples from normal semen donors were preserved by adding 10 μL of a 3 m sodium azide solution per 1 mL of the ejaculate after liquefaction. Five samples, each of 600 μL, were sent monthly to laboratories by mail during the investigation period.

### Blood samples

A blood sample was drawn from the cubital vein, centrifuged, and the serum was separated and frozen. One aliquot of each sample was sent to Denmark for a centralized hormone analysis at the University Department of Growth and Reproduction. Serum levels of follicle stimulating hormone (FSH), luteinizing hormone (LH), sex hormone-binding globulin (SHBG), testosterone and inhibin-b were measured as previously described (Andersen *et al.*, 2000). Briefly, serum levels of FSH, testosterone, SHBG and LH were determined by time-resolved-immunofluorometric assay (DELFIA, Wallac, Turku, Finland). The intra- and inter-assay coefficients of variation (CV) for measurement of FSH and LH were 3 and 4.5%, respectively. CVs for both testosterone and SHBG were <8% and <5%, respectively. Inhibin-b was determined by a specific enzyme immunometric assay, as described by Groome *et al.* (1996), with a detection limit of 18 pg/mL and intra- and inter-assay coefficients of 15% and 18%, respectively. All hormone assessments were performed at the end of the study to reduce the influence of assay variations. The free androgen index (FAI) was calculated as (Testosterone/SHBG) × 100.

### Statistical analysis

Standard statistics (mean, median, standard deviation (SD), 5–95 percentiles and frequencies) were used for description of the obtained data.

The quality control of assessment of sperm concentration did not show any intra-laboratory variation over time. The technician in the study assessed the sperm concentration at a 96% level (i.e. 4% lower) compared with the reference laboratory at University Department of Growth and Reproduction, Rigshospitalet, Copenhagen, Denmark. However, the difference was non-significant (95% confidence interval 87–106%), and thus no correction of data was made based on the results of the quality control.

Multiple regression analyses were carried out to obtain adjusted semen and hormonal values corrected for co-variates. Semen volume, sperm concentration, total sperm counts and reproductive hormone values were normalized by natural logarithmic transformation to correct for a skewed distribution. The percentages of motile spermatozoa were logit-transformed, whereas the percentages of morphologically normal spermatozoa were analysed untransformed.

The duration of abstinence up to 96 h had an increasing effect on semen volume, sperm concentration and total sperm count (all *p* < 0.05). For percentages of motile spermatozoa, the duration from ejaculation to assessment of motility was evaluated as an additional confounder, but found to be non-significant. However, the estimated values indicated decreasing motility when evaluations were performed later than 30–60 min after ejaculation, and thus duration from ejaculation to assessment was included as confounder in the statistical model. The duration was also tested as a confounder for the other semen variables, but found to be non-significant and therefore not included in the model for these variables. The observer in Finland assessed 235 of the 256 morphology slides, and was used as reference in the statistical model to correct for the observer variation between the two investigators. The two observers entered the regression model as a dummy variable. None of the potential confounders had any effect on morphology. No effect of season of year, age of the men or collection site (at home vs. at study site) could be detected for the semen variables. Hour of blood sampling had a decreasing effect on inhibin-b and testosterone and was included as confounder in the regression analyses for the hormones. The final models were subjected to standard checks of the residuals. The statistical analyses were performed using PASW (GradPack 18.0; SPSS Inc., Chicago, IL, USA).

## Results

The basic description of the study population is summarized in Table 1. Among the 273 men included in the study, twenty men had not answered the questionnaire prior to participation in the study as requested, and did not hand it in later, although they agreed upon during the investigation. Thus, the questionnaire information in Table 1 is based on 253 men. The participation rate to the study was 78% for the men who received information at the University of Almeria ‘Open Days’.

**Table 1:** Physical appearance and self-reported information of young men from the Almeria province in Spain

	Mean (SD)	Median (5–95)
Height (cm)	177.7 (6.4)	178.0 (167.3–188.8)
Weight (kg)	75.3 (10.5)	75.0 (60.0–94.0)
BMI (kg/m^2^)	23.8 (3.0)	23.7 (19.6–29.4)
Size, left testis (mL)^a^	18.4 (4.7)	20.0 (12.0–25.0)
Size, right testis (mL)^a^	18.6 (4.5)	20.0 (12.0–25.0)
Mean of left and right size (mL)^a^	18.4 (4.6)	20.0 (12.0–25.0)
Age (years)^b^	21.3 (2.1)	20.9 (18.4–24.9)
Ejaculation abstinence (hour)^c^	75 (40)	67 (35–156)
Frequency (%)		

Have (had)		
Cryptorchidism^d^	8.7	
Cryptorchidism, treated^e^	3.6	
STD diagnosed^f^	1.6	
Varicocele diagnosed	0.4	
Inguinal hernia diagnosed	1.2	
Prolonged disease^g^	6.7	
Diabetes or thyroid disease	0.4	
One or more of the above stated	16.3	
Good general health^h^	98.4	
Taken medicine^i^	34.0	
Smokers	32.8	
Cigarette smokers	32.8	
Marijuana/hashish smokers	22.1	
Exposed to tobacco in utero^j^	5.9	

SD, standard deviation.

(5–95): 5–95th percentile.

a Size assessed by palpation. Two men had non-palpable right testicles, one due to previous orchidectomy because of cancer and one for unknown reasons.

b Age calculated as difference between day of attendance in study and self-reported day of birth.

c Ejaculation abstinence period calculated as difference between time of current ejaculation and self-reported time of previous ejaculation.

d Not born with both testicles in scrotum (irrespective of spontaneous descend, treatment or still cryptorchid).

e Hormonal, surgical or combination.

f Diagnosed with epididymitis, chlamydia or gonorrhoea.

g Question was ‘Have you ever had any long-lasting disease?’.

h Question was ‘How will you describe your own health? Good, fair or poor’.

i Taken any medication recent 3 months prior to participation in study. For 85%, it was either antibiotics or against allergy.

j In utero exposed to maternal tobacco smoking.

In satisfying the inclusion criteria, all men lived in the Almeria province: 85.5% in Almeria city (approximately 200 000 inhabitants) and the remaining 14.7% in the surrounding smaller cities (each <10 000 inhabitants). Most men (89.3%) were university students, 6.7% had finished high school and 4.0% had primary school as their highest completed school education.

Majority of men (98.4%) considered themselves to have a good general health; however, approximately one-third had taken some kind of medication during the 3 months before participation in the study, and among these it was mainly ‘over-the-counter’ antibiotics or medicine against simple allergies. Of the men, 32.8% were cigarette smokers, and among these, 67% also reported use of marijuana.

Almost 9% reported that they were not born with both testicles in scrotum and 3.6% had been treated for the condition. Altogether, 16.3% of men, prior to participation in the study, had one or more of the following: Cryptorchidism, epididymitis, chlamydia, gonorrhoea, varicocele, inguinal hernia or prolonged disease including diabetes or thyroid disease. The median testis size was 20 mL as assessed by the orchidometer (Table 1), and 98.5% had normal adult pubic hair distribution. Ten per cent were found to have a varicocele.

The investigation period ranged from November 6, 2001 to December 2, 2002; 27.1% of men were investigated during March–May, 7.7% June–August, 48.4% September–November and 16.8%, December–March. The median duration of ejaculation abstinence was 67 h (Table 1), including 7% of men having <48 h of abstinence.

Semen results are summarized in Table 2. The ‘Observed’ values are based on raw data, and the ‘Adjusted’ are the calculated expected values correcting for confounders. For semen volume, sperm concentration and total sperm count, the values are adjusted to an ejaculation abstinence period of 96 h, sperm motility to assessment 30–60 min after ejaculation. The adjusted median sperm concentration and total sperm count were 62 million/mL (95% CI 47–82) and 206 million (95% CI 153–278), respectively (Table 2). Twelve per cent and 27%, respectively had sperm concentration below 20 × 10^6^/mL and 40 × 10^6^/mL. A total of 21, 52 and 68% had less than 5, 9 and 12% morphologically normal spermatozoa and 25% less than 50% motile spermatozoa. Morphology results were based on samples from only 256 men. Some slides were broken when received in Turku, Finland, for morphology analyses. However, some had previously been assessed in Almeria. When combined, the morphology results are therefore based on 235 slides assessed in Turku and 21 in Almeria. Table 2 also shows the adjusted values for the subgroup of men who had not taken any medicine and were without any previous or current andrological diseases.

**Table 2:** Semen quality of young men from the Almeria province in Spain

	Observed – All men		Adjusted – All men	Adjusted – Subgroup
			
	Mean (SD)	Median (5–95)	Median (95% CI)	Median (95% CI)
Semen volume (mL)	3.1 (1.5)	3.0 (1.0–5.8)	3.4 (3.0–3.7)	3.4 (3.0–3.8)
Sperm concentration (million/mL)	72 (70)	51 (5–206)	62 (47–82)	75 (54–102)
Total sperm count (million)	215 (240)	149 (8–599)	206 (153–278)	246 (179–338)
Motile spermatozoa (%)	59 (16)	60 (28–85)	59 (55–63)	61 (58–64)
Morphologically normal forms (%)^a^	9.4 (5.5)	8.3 (1.9–20.6)	9.4 (8.7–10.2)	9.6 (8.7–10.5)

Observed: Results based on raw data.

SD, standard deviation.

(5–95): 5–95th percentile.

Adjusted median and 95% CI (confidence interval) calculated by linear regression analysis. Sperm concentration, total sperm counts and semen volume adjusted to a period of ejaculation abstinence of 96 h. Motility controlled for duration from ejaculation to assessment. Morphology adjusted for inter-observer variation. See text for further explanation.

All men: Results based on all men participating in the study.

Subgroup: Results entirely based on the 60.5% of the study population not taking any medication, men without any previous or current andrological diseases including known fertility problems. Information obtained from self-administered questionnaire.

a Morphology results only available for 256 men.

The 5.9% of the sons who had been exposed to maternal smoking during their mother’s pregnancy had significantly reduced median sperm concentration and total sperm counts; 44% (95% CI 24–79%, *p* = 0.006) and 50% (27–93%, *p* = 0.03) of non-exposed, respectively. No effect of in utero exposure to maternal smoking could be detected for motility or morphology. Men’s own smoking habits did not affect any of the semen variables (all *p* > 0.2), when adjusted for the effect of in utero exposure. Men born in the smaller cities of the Almeria province tended to have higher total sperm counts and sperm concentrations (*p* = 0.05 and *p* = 0.17) than men born in the Almeria city. Twenty-nine per cent of the mothers were born in rural areas, but this did not influence the son’s semen variable when correcting for his place of birth. Season of investigation, men’s age, BMI, medication in the recent 3 months, previous prolonged disease or recent alcohol intake did not influence the semen variables (all *p* > 0.05).

Table 3 summarizes the serum hormone levels of the participants. The adjusted values are corrected for time of day of blood sampling to represent sampling at 10 am. All men had detectable levels of the assessed hormones.

**Table 3:** Reproductive hormones of young men from the Almeria province in Spain^a^

	Observed – All men		Adjusted – All men	Adjusted – Subgroup
			
	Mean (SD)	Median (5–95)	Median (95% CI)	Median (95% CI)
FSH (U/L)	3.1 (1.7)	2.8 (1.1–6.5)	2.5 (2.2–2.8)	2.4 (2.1–2.8)
Inhibin-b (pg/mL)	174 (67)	164 (80–280)	160 (144–177)	174 (154–196)
LH (U/L)	4.1 (1.8)	3.8 (1.7–7.6)	3.7 (3.4–4.1)	3.8 (3.4–4.2)
Testosterone (nm)	25 (8)	24 (13–40)	28 (26–30)	28 (26–31)
SHBG (nm)	30 (10)	30 (14–48)	29 (27–31)	29 (26–31)
FAI^b^	90 (39)	82 (42–166)	95 (88–103)	99 (90–100)

Observed: Results based on raw data.

SD, standard deviation; FAI, free androgen index; FSH, follicle stimulating hormone; LH, luteinizing hormone; SHBG, sex hormone-binding globulin.

(5–95): 5–95th percentile.

Adjusted median and 95% CI (confidence interval) calculated by linear regression analysis, adjusted to blood sampling at 10:00 am.

All men: Results based on all men participating in the study.

Subgroup: Results entirely based on the 60.5% of the study population not taking any medication, men without any previous or current andrological diseases including known fertility problems. Information obtained from self-administered questionnaire.

a Serum samples were only available for 250 of the 273 participants. Thus, hormonal results are based on these 250.

b Calculated free androgen index.

The sperm concentration and total sperm counts of the 20 men who did not return the questionnaire were only 49% (95% CI 28–83%) and 47% (95% CI 27–83%), both *p* = 0.009, of the values of those returning it. Their testicles were, on average, 2.6 mL smaller (*p* = 0.02). Their inhibin-b (89%, *p* = 0.3) and testosterone (94%, *p* = 0.4) levels did not differ significantly from those of others. There was no tendency for any difference regarding their age, height, weight, BMI, ejaculation abstinence period or any other semen or hormone values. We did not exclude these men because they were otherwise eligible to the study.

**Figure 1:**
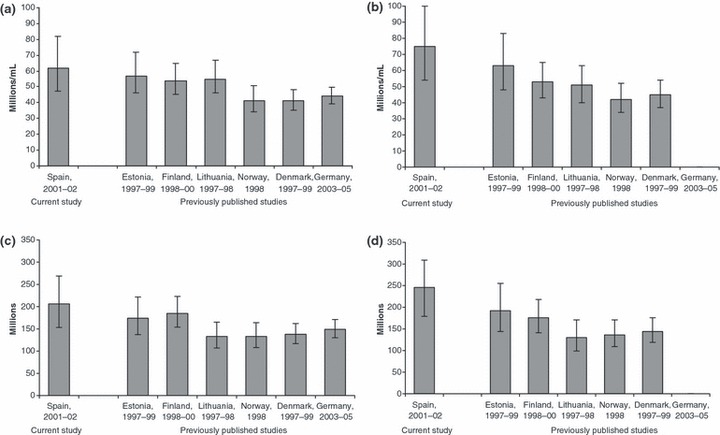
Sperm counts of young men from a south-eastern area of Spain shown together with previously published results from six North-European countries (Jørgensen *et al.*, 2002; Punab *et al.*, 2002; Paasch *et al.*, 2008). The bars show the adjusted median sperm concentration (million/mL) in the entire study populations (a) and in subgroups of men without any recent use of medicine or known andrological disease in their history (b), and total sperm count (mill) for the entire groups (c) or subgroups (d). Black, vertical lines show 95% confidence intervals of the estimated medians. A combined estimate for the German subgroup has not been published, and is therefore not included. The period of investigation of the studies is shown at the *X*-axis.

Figure 1 shows the estimated, adjusted sperm concentration and total sperm count for the Spanish men together with previously published results for Estonian, Finnish, Norwegian, Danish (Jørgensen *et al.*, 2002), Lithuanian (Punab *et al.*, 2002) and German men (Paasch *et al.*, 2008). These previously published studies had all utilized the same study protocol as the current study as described in the methods section, and all the study centres participated in the same quality control programme for assessment of sperm concentration.

## Discussion

Our study population of young men from the Almeria province in Spain had semen quality and reproductive hormone levels as expected in a normal population. A direct statistical comparison with men from the northern European countries was not performed. However, the Spanish men seemed to have higher sperm concentrations, total sperm counts (as illustrated in Fig. 1) and testosterone levels than previously reported for men from the northern European countries (Jørgensen *et al.*, 2002; Punab *et al.*, 2002; Paasch *et al.*, 2008).

The investigation procedures in our study were similar to those used in the northern European studies. The evaluation of sperm concentration was controlled by an external quality control study and all hormonal analyses had been performed in the same laboratory. Whether the men collected the semen at home or at the study site did not influence the results. In addition, the same types of statistics were applied in all studies, including adjustment for the effect of confounding factors. Therefore, technical aspects do not hamper comparisons of sperm concentration and total sperm counts between these studies. Sperm morphology was assessed according to strict criteria in all the studies. Ideally, all morphology slides should have been assessed by only one investigator. Unfortunately, some glasses were broken when received in the Finnish laboratory and could not be assessed there. However, a majority of slides had at that time already been assessed in Spain, and we chose to utilize these results, and correct for the different observers in the statistical analyses. Motility assessment is highly influenced by inter-observer variation (Jørgensen *et al.*, 1997), and we did not have any quality control programme for this. Therefore, we hesitate to draw any firm conclusions regarding the motility findings.

In the interpretation of our results, it is important to discuss whether our study population is comparable to those published for the Nordic-Baltic countries. In the Nordic-Baltic countries and Germany, all young men, except those suffering from chronic severe disease, are required to undergo a compulsory medical examination to determine their fitness for military service. Such an examination process was given up in Spain in 1999 when military service became voluntary. Therefore, we had to include our study subjects differently as described in the Materials and methods section. An important question to consider is therefore whether selection bias may play a role (Eustache *et al.*, 2004). We tried to avoid this first by approaching men of almost similar age and requiring the same basic birthplace criteria as in the studies in the northern Europe. Secondly, we tried to overcome selection of men with either particularly good or bad semen quality by excluding men who previously had caused a pregnancy, had been trying, or previously had semen analysed. Therefore, any prior knowledge of own fertility potential is unlikely to have affected their motivation to participate. Concern about fertility potential is unlikely to have caused a severe bias in our study because the proportion of men with urogenital diseases in their history was very low. The studies in the northern Europe did not require that the men should be without direct knowledge of their testicular function. The modest financial compensation (20 Euros) is unlikely to have introduced any systematic bias. Thus, we find it feasible to include a well-defined study cohort, which can be considered a reference group for future studies investigating temporal changes.

It is obvious that our study group mainly reflects men with a high educational level. In 2002, 69% of all young men from Almeria province were university students (http://www.juntadeandalucia.es), and thus university students were over-represented in our study (89% of participants). Higher educational level has previously been positively associated with semen quality (Jensen *et al.*, 2001; Eustache *et al.*, 2004; Muller *et al.*, 2004).

The Spanish men were 18–25 years old and in average approximately 2 years older than those examined in the Nordic-Baltic countries. We did not see any effect of age when including this as a co-variate in the regression analyses. Furthermore, a 4-year follow-up study of young Danish men also showed that their semen quality did not increase with increasing age (Carlsen *et al.*, 2005). The age difference is therefore not likely to explain the different semen quality levels between the Spanish and the northern European men.

The studies from the Nordic-Baltic countries also described a subgroup of men without any andrological diseases in their current or past history or intake of medicine (Jørgensen *et al.*, 2002; Punab *et al.*, 2002), and comparing the Spanish results with these subgroups does not change the interpretation of the Spanish results. Thus, we believe, the results from our study provide reliable information of the semen quality of young men from the southern part of Spain despite the potential selection problems described above.

To our knowledge, only one study that aimed to investigate men from the general Spanish population has been published (López-Teijón *et al.*, 2008). This study summarized results from more than 40 centres, and included 1239 men among which almost 700 were semen donor candidates. In our interpretation, it was not excluded that unknown selection bias could have influenced the results of that study. Nevertheless, it is striking that it also showed a relatively high mean and median sperm concentration (unadjusted 68.8 and 60.0 million/mL, respectively) and that men from the Andalucía region, which also include the Almeria province where our study was undertaken, had one of the highest sperm concentrations.

Previous studies on secular trends in male reproductive health provided conflicting results. Some suggested that sperm counts had declined significantly, whereas other did not find evidence of any change (Carlsen *et al.*, 1992; Auger *et al.*, 1995; Bujan *et al.*, 1996; Irvine *et al.*, 1996; Paulsen *et al.*, 1996; Van Waeleghem *et al.*, 1996; Vierula *et al.*, 1996; Swan *et al.*, 1997). A feature was, however, the appearance of regional differences in semen quality, which were as great as the suggested secular trend. However, the interpretation of these retrospectively designed studies may have been influenced by selection bias because they dealt with highly selected groups of men (Jouannet *et al.*, 2001). Nevertheless, a French study of fertile men more clearly indicated the existence of regional differences in human semen quality within France (Auger *et al.*, 1997), and later differences between fertile men from four European countries and four sites in the US were also detected (Jørgensen *et al.*, 2001; Swan *et al.*, 2003). Furthermore, publications have also shown regional differences in semen quality among young men not selected because of their fertility (Jørgensen *et al.*, 2002; Punab *et al.*, 2002; Richthoff *et al.*, 2002; Paasch *et al.*, 2008). In this perspective, the present study did not only show the investigated men to have a normal semen quality; it also added further evidence to the existence of geographical differences in semen quality between young men from various populations.

The finding of higher sperm counts in Spanish men is in agreement with our hypothesis based on the TDS concept that associate impaired semen quality with an increased risk of testicular cancer and vice versa. The incidence of testicular cancer has increased in most industrialized countries during the past 40–50 years (Richiardi *et al.*, 2004; Bray *et al.*, 2006; Huyghe *et al.*, 2007). The incidence of testicular cancer in the Nordic-Baltic region follows the same east-west gradient as semen quality in this area. Despite that, testicular cancer incidence is also increasing in Spain, the absolute risk is still very low (incidence rate of 1.91/100 000 men in 2005 in the Madrid area) (Llanes González *et al.*, 2008) compared with the northern European countries (Denmark 9.2, Norway 9.6 and Germany (Saarland) 8.1/100 000 in the period 1998–2002) (Chia *et al.*, 2010), and also lower than among Finns (4.3/100 000, 2001–2005) (Finnish Cancer Registry, 2009).

The TDS concept also suggests that an impaired development of foetal testes could lead to an increased risk of decreased spermatogenesis. Our finding of reduced semen quality among men who prenatally were exposed to maternal tobacco smoking is consistent with this. Similar effects of in utero exposure to tobacco smoking have been shown in other European populations (Jensen *et al.*, 2004, 2005a,b; Paasch *et al.*, 2008).

Prenatal adverse events do not exclude that exposures later in life may also contribute to impairment of semen quality (Hauser *et al.*, 2005; Bonde *et al.*, 2008; Jørgensen *et al.*, 2010). Whether the lower than expected frequency of motile and morphologically normal spermatozoa in our study population can be attributed to any current exposure remains to be explored. We did not have any direct measures of current chemical exposures, but only a few information of current lifestyle (tobacco smoking, recent alcohol intake or recent use of medicine) that did not affect the evaluated semen variables significantly. Furthermore, we do not have any data available that can elucidate to which degree the observed levels of the semen variables of the young Spanish men are partly due to a special genetic background among these men leading to normal spermatogenesis. From studies of testicular cancer risk in American men, it is clear that testicular cancer risk is partly dependent on genetics as the risk among Black American is lower than the risk for White Americans, despite living in the same areas (McGlynn *et al.*, 2005). We cannot exclude that a similar aspect may apply to the testicular cancer risk and function of testicles of Spanish men.

## Conclusion

We have shown that the young men from the Southern Spain have normal semen quality and reproductive hormone levels as expected in a population with low testicular cancer risk. The Southern Spain has experienced an increasing industrialization and modernization in agriculture practices in the recent years and with this, an increased risk of adverse exposures, and only follow-up studies of new cohorts in the future will be able to determine whether the testicular function of Spanish men becomes affected.
